# Location-Specific Predictors of Double Burden of Malnutrition among Nigerian Mother–Child Pairs: Re-evaluating Dietary Quality and Socioeconomic Factors

**DOI:** 10.1016/j.tjnut.2026.101478

**Published:** 2026-03-12

**Authors:** Beulah F Ortutu, Faidat A Adeleke, Hajara Idris, Chiamaka J Ezenwa, Folasade A Adeleke, Okemudi Nwonye, Linda O Edafioghor, Ifeoma M Egechizuorom, Gideon O Iheme

**Affiliations:** 1 Department of Nutrition, Texas A&M University, TX, United States; 2 Department of Human Nutrition and Dietetics, Michael Okpara University of Agriculture, Umudike, Nigeria; 3 Department of Human Nutrition and Dietetics, University of Ibadan, Ibadan, Nigeria; 4 Department of Pediatrics, Federal Teaching Hospital Katsina, Katsina, Nigeria; 5 Department of Public Health, Federal University of Technology, Owerri, Nigeria; 6 Department of Nutrition and Dietetics, Modibbo Adama University Teaching Hospital, Yola, Nigeria; 7 Department of Nutrition and Dietetics, Federal University of Agriculture, Mubi, Nigeria; 8 Department of Nutrition and Dietetics, Federal University of Medical and Health Science, Kwale, Delta state, Nigeria; 9 Department of Nutrition and Dietetics, Federal Medical Centre, Umuahia, Nigeria; 10 Department of Food Studies, Nutrition and Dietetics, Uppsala University, Uppsala, Sweden

**Keywords:** double burden of malnutrition (DBM), mother–child pairs, wealth index, food insecurity, diet quality, maternal education, Nigeria

## Abstract

**Background:**

The double burden of malnutrition (DBM), classified as the coexistence of maternal overweight/obesity or maternal undernutrition and child undernutrition or childhood overweight/obesity, within the same household, is an increasing concern in Nigeria. Drivers of DBM may differ by location due to urbanization, socioeconomic gradients, and dietary transitions.

**Objectives:**

This study examined location-specific predictors of DBM among Nigerian mother–child pairs, with a focus on child dietary quality, maternal education, household food insecurity, and wealth index.

**Methods:**

A descriptive cross-sectional study using a stratified multistage sampling technique was conducted among 1295 mother–child pairs (children aged 6–23 mo) across 4 Nigerian cities. Child nutritional status was assessed using World Health Organization (WHO) growth standards, and maternal body mass index was classified according to WHO adult cutoffs. Household food insecurity, dietary diversity, minimum meal frequency, and wealth index were measured using validated tools. Associations between predictors and DBM were examined using χ^2^ tests and generalized estimating equations, including interaction and stratified analyses by location.

**Results:**

DBM prevalence was 37.4%, with the most frequent phenotype being overweight mothers and undernourished children (34%). Semiurban residence [adjusted odds ratio (AOR): 2.11; 95% confidence interval (CI): 1.93, 2.30], food-secure households (AOR: 1.22; 95% CI: 1.09, 1.37), and not meeting the minimum meal frequency (AOR: 1.41; 95% CI: 1.04, 1.92) were associated with an increased risk of DBM.

**Conclusions:**

DBM among Nigerian mother–child pairs is shaped by dietary factors. Context-specific interventions are needed, with a focus on improving child diet quality in semiurban areas.

## Introduction

The nutritional landscape in low- and middle-income countries has become complex, with increasingly public health concerns surrounding the double burden of malnutrition (DBM), especially among mother–child pairs [1,2]. DBM is the coexistence of maternal overweight/obesity and child undernutrition (stunting, wasting, or underweight) or maternal undernutrition and child overweight/obesity within a household [3]. In these countries, nutrition transition shifts dietary patterns toward the consumption of energy-dense, ultraprocessed foods [4], contributing to a sharp rise in overweight and obesity in a household, whereas undernutrition remains prevalent within the same household [5,6]. Nigeria faces a substantial burden of both forms of malnutrition: recent data show that 33.8% of children under 5 are stunted, 11.5% are wasted, 25.5% are underweight, and 1.5% are overweight, whereas among females of reproductive age, 15% are overweight and 8.1% are obese [7]. These statistics reflect a national nutrition crisis and a potential for DBM to arise from a more profound structural and behavioral shift.

The DBM is driven by multifaceted determinants, including access and availability of nutritious food, food insecurity, poverty, educational status, urbanization, and lifestyle changes [3,8,9]. However, existing literature has consistently positioned poverty and food insecurity as central risk factors in DBM, often simplifying interventions to focus mainly on economic strengthening and the distribution of food aid [[10], [11], [12]]. Several studies have repeatedly revealed strong associations between DBM and low wealth index and food insecurity [11,[13], [14], [15], [16]]. However, these traditional risk models may overlook the impact of urbanization and nutrition transition in low- and middle-income countries, such as Nigeria, where dietary quality may have stronger influences on DBM risk than poverty-related indicators [17,18]. Despite these findings, evidence remains limited on how socioeconomic and dietary factors influence DBM risk within the same household across urban and semiurban areas in Nigeria. Few studies have explored whether dietary quality now plays a stronger role than the traditional economic indicators [17,18].

As Nigerian households undergo rapid urbanization, simple economic measures may fail to capture the complexity of education, religion, ethnicity, culture, and individual choices influencing dietary behaviors and patterns. In this context, it is crucial to reassess whether economic hardship and household shortage remain the most decisive predictors of DBM, or whether new, location-specific determinants are emerging. Therefore, this cross-sectional study aims to investigate the socioeconomic, dietary, and maternal-related factors associated with DBM among mother–child pairs in Nigeria. We examined key indicators, including food insecurity, wealth index, maternal education, and child dietary quality. We also explored interaction effects and stratified analyses by residential location to capture urban-semi-urban differences in DBM risk.

## Methods

### Study design

This study used a descriptive cross-sectional design aimed at investigating the DBM among mother–child pairs across 4 geographic areas in Nigeria. This design was selected for its suitability in providing a snapshot of nutritional status and associated household factors at a specific point in time, allowing for the identification of relationships between socioeconomic status, household food insecurity, and nutritional outcomes without the complexity of longitudinal follow-up.

### Study settings

This study was conducted across 4 geographically and culturally diverse cities in Nigeria, representing different geopolitical zones: Umuahia, Abakaliki, Yola, and Katsina. These locations were strategically selected to reflect a range of sociodemographic and environmental contexts relevant to child health and nutrition. Although all state capitals are administratively classified as urban, Katsina (Katsina State) and Yola (Adamawa State) are relatively more developed urban centers, with denser populations and greater access to commercial and educational infrastructure [19]. In contrast, Abakaliki (Ebonyi State) and Umuahia (Abia State) are less developed and are often described as semiurban settings, with comparatively more limited access to certain health and social services.

On the basis of these characteristics and established regional placement, the study population was stratified into urban/northern (Yola and Katsina) and semiurban/southern (Abakaliki and Umuahia) groups, ensuring relevance to the study objectives by capturing a range of socioeconomic conditions and dietary transitions, thereby enhancing the representativeness of the sample in these diverse Nigerian contexts. Data collection was carried out over 3 mo, from February to April 2024, within pediatric-focused settings where mother–child pairs routinely accessed services, ensuring relevance to the study objectives and the inclusion of a representative sample.

### Population and sampling

The study population comprised mother–child dyads with children aged between 6 and 23 mo. This age range was selected because it represents a critical window for nutritional vulnerability, encompassing the period of complementary feeding transition and rapid growth. Mothers eligible for inclusion were primary caregivers who willingly consented to participate in the study. Dyads were excluded if the child presented with congenital abnormalities or chronic illnesses that could independently affect nutritional status, or if the mother was unable to provide reliable information due to cognitive impairment or severe illness. Participant recruitment was carried out using a stratified multistage sampling approach. First, the study sites were purposively stratified by geographic and urbanization characteristics into northern/urban (Yola and Katsina) and southern/semiurban (Abakaliki and Umuahia) locations. Within each city, community immunization centers were identified as primary recruitment zones because they serve as routine points of contact for mothers with young children. Where multiple immunization centers were available within a city, centers were selected to ensure coverage across different catchment areas.

**FIGURE 1:**
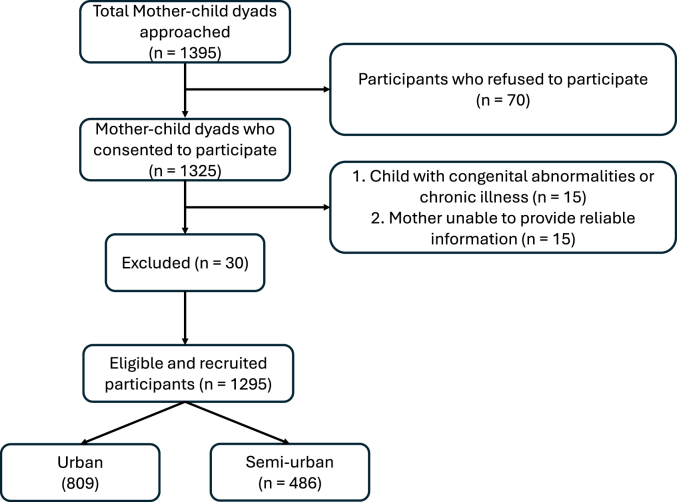
Flow chart of participant selection.

Within selected immunization centers, participant recruitment followed a consecutive sampling approach, whereby all eligible mothers attending the centers during the study period were invited to participate. This approach allowed for efficient recruitment while maintaining the heterogeneity of the sample population and reflecting real-world community settings. A total of 1295 mother–child dyads were recruited across the 4 study sites. The sample size was determined by the number of eligible and consenting participants identified during the recruitment period, rather than by a priori calculation (Figure 1). This approach ensured complete enumeration of the accessible population within the defined sampling frame and maximized representativeness of mother–child pairs attending community immunization centers.

All eligible mothers who visited the selected immunization centers during the study period were invited to participate after the study objectives were explained. Participation was voluntary, and only those who provided informed consent were recruited. The response rate was high, as most eligible mothers agreed to participate after being approached. However, because recruitment was restricted to mothers who attended immunization services, the sample may overrepresent females who are more health-conscious, educated, or of higher socioeconomic status, which could significantly limit the generalizability of these findings to broader, less engaged populations. A post hoc power analysis was conducted to evaluate the adequacy of the achieved sample size. With a total sample of *n* = 1295 at *α* = 0.05, the power to detect a small effect (Cohen’s *f*^2^ = 0.02) exceeded 0.99. The power to detect a medium effect (*f*^2^ = 0.15) was also >0.99. These results indicate that the sample size was sufficient to detect small-to-moderate associations in the multivariable analyses.

### Ethical considerations and informed consent

Ethical approval for this study was obtained from the Research and Ethics Committees of health institutions located within the study cities, namely: Federal Medical Center, Umuahia (FMC/QEH/G.596/Vol.10/576); Modibbo Adama University Teaching Hospital, Yola (FMCY/SUB/S.128/161); Alex Ekwueme Federal University Teaching Hospital, Abakaliki (AE-FUTHA/REC/VOL.3/2022/002); and Federal Teaching Hospital, Katsina (FTHKNREC.REG.25/06/22c/017). Though data collection was conducted at the community level, these institutions provided oversight and ethical clearance relevant to each geographical region. The study was conducted in full accordance with the ethical principles outlined in the Declaration of Helsinki and Nigeria’s National Code for Health Research Ethics, ensuring respect for participants’ dignity, safety, and autonomy.

Informed consent was obtained before any data collection activities commenced. Mothers or primary caregivers provided written informed consent using either paper-based forms or electronic consent administered on-site at the immunization centers using digital devices. For electronic consent, participants indicated agreement by checking a mandatory “I agree” box before proceeding with the questionnaire. Confidentiality and anonymity were strictly maintained throughout the study. All data collected were deidentified, coded, and securely stored in password-protected systems, accessible only to the research team. No personal identifiers were used in any data analysis, reporting, or publication.

Every effort was made to minimize discomfort and ensure participants' safety, including the use of trained data collectors for anthropometric measurements and ethical handling of sensitive topics such as household food insecurity. No invasive procedures were involved. The research team remained available throughout the study to address any concerns from participants.

### Study instruments and data collection

Data collection was conducted by a team of trained health professionals who administered structured questionnaires and performed anthropometric assessments in accordance with standardized protocols. The questionnaires, developed from validated instruments and pretested for clarity and cultural appropriateness, gathered detailed information on household demographics, asset ownership, and food insecurity experiences. Anthropometric measurements were conducted with calibrated equipment to ensure accuracy. Children’s weight was measured using electronic Salter scales to the nearest 0.1 kg, with younger infants weighed naked and older children lightly clothed, whereas length or height was measured to the nearest 0.1 cm using infantometers or stadiometers, depending on age and ability to stand. Mothers’ weight and height were measured using standardized bathroom scales and stadiometers, respectively, with measurements taken twice to enhance reliability, and the average was used in the BMI (in kg/m^2^) calculation [20].

Efforts to reduce bias included rigorous training of data collectors to standardize procedures, supervision by experienced nutritionists, and double data entry to minimize input errors. The selection of multiple sites within the community hubs enhanced the external validity and generalizability of findings. Additionally, the exclusion of children with conditions known to affect nutritional status minimized confounding.

### Variables

The primary outcome of interest was the DBM, defined as the coexistence of child undernutrition and maternal overweight or obesity, or the coexistence of childhood overweight or obesity and maternal undernutrition within the same household. Child nutritional status was assessed using standardized anthropometric indices: weight-for-age (WAZ), height-for-age (HAZ), weight-for-height (WHZ), and BMI for age (BAZ) *z-*scores, calculated based on the WHO Child Growth Standards. Children with *z-*scores <−2 SD in any of these indices were classified as undernourished, specifically underweight (WAZ <−2), stunted (HAZ <−2), or wasted (WHZ <−2), whereas children with *z*-scores above +2 were classified as being overweight [21]. The WHO Anthro Survey Analyzer tool (version 1.0.3) was used to compute the *z*-score [22]. Maternal nutritional status was evaluated using BMI, derived from measured weight and height, and categorized according to WHO adult BMI classifications: underweight (<18.5), normal (18.5–24.9), overweight (25.0–29.9), and obese (≥30.0) [23]. A household was classified as experiencing the DBM if ≥1 child was undernourished, and the mother was overweight or obese (≥25). DBM could only be computed for dyads with complete anthropometric measurements for both mother and child; therefore, missing data for DBM primarily arose from instances where one or both anthropometric measurements were not taken due to practical limitations, indicating structural nonmeasurement rather than nonresponse.

To assess the household context influencing DBM, 5 explanatory variables, including household food insecurity, minimum meal frequency (MMF), dietary diversity scores (DDS), and socioeconomic status, were evaluated. Household food insecurity was measured using the Household Food Insecurity Access Scale (HFIAS), a validated tool developed by the Food and Nutrition Technical Assistance Project (FANTA). The HFIAS consists of 9 occurrence questions assessing anxiety and uncertainty about food supply, insufficient quality, and insufficient food intake, and its physical consequences. Responses were scored based on frequency of occurrence in the past 4 weeks: never (0), rarely (1), sometimes (2), and often (3). The total HFIAS score ranges from 0 to 27, with higher scores indicating more severe food insecurity. On the basis of FANTA guidelines, households were categorized into 4 levels of food security: food secure (0–1)—depicting minimal or no worry about food, mildly food insecure (2–7)—characterized by occasional worry or reliance on less preferred food alternatives with no quantity reduction, moderately food insecure (8–14)—demonstrated by substantial reductions in food quality and quantity, and severely food insecure (15–27)—referring to persistent food reductions potentially leading to complete food absence for a specific period or even an entire day [24].

In accordance with WHO/UNICEF (2021) guideline, MMF was defined as the proportion of breastfed and nonbreastfed children aged 6–23 mo who receive solid, semisolid, or soft foods (but also including milk feeds for nonbreastfed children), for a minimum of 2 times for breastfed infants aged 6–8 mo, or 3 times for breastfed children aged 9–23 mo, or 4 times for nonbreastfed children 6–23 mo. If the child achieved the MMF, it was coded as “yes =1,” whereas if the child did not achieve the MMF, it was coded as “no = 0” [25,26].

DDSs were assessed using standards established by the FAO [27]. Foods groups were categorized into 8 groups: *1*) grains, roots, and tubers, *2*) legumes and nuts, *3*) dairy products, *4*) fleshy foods (meat, fish, poultry, liver, and organ meat), *5*) eggs, *6*) vitamin A–rich fruits and vegetables, *7*) other fruits and vegetables, *8*) sweet beverages. The additional group “sweet beverages” was included because these beverages are widely consumed in this population and contribute substantially to energy intake and feeding practices among young children. Previous studies in West African settings have incorporated similar groups to account for sugary drinks and processed snack foods [28]. We categorized DDS as low (0–2 food groups), medium (3–4 food groups), and high (≥5 food groups). A score ≥5 corresponds to achieving the WHO/UNICEF Minimum Dietary Diversity (MDD) for children aged 6–23 mo [26]. Participants receive 1 point for consuming any food group from the above categories within the last 24 h prior to questionnaire administration. If a food group is not consumed, they receive 0 points.

Socioeconomic status was approximated using a Household Wealth Index (HWI), constructed through Principal Component Analysis of variables related to household assets (e.g., ownership of radio, refrigerator, and mobile phone), housing quality (e.g., flooring material), and access to services (e.g., electricity, water source, and toilet facility). The first principal component explained 23.1% of the total variance and was used as the socioeconomic index. The full component loadings are presented in Supplementary Table 1. After the approach used in Demographic and Health Surveys, the resulting wealth scores were used to classify households into tertiles or quintiles (e.g., poorest, middle, and richest), thereby facilitating analysis of socioeconomic gradients in nutritional outcomes [29].

### Statistical analysis

Quantitative variables such as anthropometric indices, HFIAS scores, and wealth index scores were analyzed both as continuous and categorical variables to facilitate interpretation and comply with established cutoffs. The WHO Anthro software version 3.2.2 was used to compute *z*-scores for child anthropometry, whereas maternal BMI categories adhered to WHO standards. Statistical analyses were conducted using SPSS version 29 (IBM SPSS Statistics), where descriptive statistics characterized the study population. The χ^2^ tests were used to examine relationships between malnutrition and household factors. To control potential confounders and explore independent predictors of the DBM, generalized estimating equations (GEE) models were fitted. Because of the absence of a prior sample calculation, we conducted a post hoc power analysis using the univariate linear regression power module as a conservative approximation for the binary logistic analyses. An interaction model to account for possible synergistic effects between overall DBM, maternal education, wealth index, and dietary intake markers using binary logistic regression analysis. Interaction terms were evaluated to identify effect modifiers, and model fit was assessed through standard diagnostics. A *P* value <0.05 was considered statistically significant. We examined the extent and pattern of missingness using Missing Value Analysis. Missingness was concentrated in DBM (24.2%). Little’s MCAR (Missing Completely At Random) test indicated that missingness was *P* = 0.455. Complete-case DBM analyses yielded stable estimates; therefore, missing data were processed using pairwise deletion.

## Result

### Sociodemographic characteristics of participants

A total of 1295 females with children <23 mo of age participated in this study. The sociodemographic and socioeconomic characteristics of the study participants are described in Table 1. Food security was assessed using the household food insecurity assessment scale scores presented in Supplementary Table 2. The majority of the children had low dietary diversity, and >50% did not meet the MMF. Dietary diversity food group classification is presented in Supplementary Table 3.

**TABLE 1:** Sociodemographic characteristics of households.

Variables	Frequency	Percentage (%)
Umuahia	189	14.6
Katsina	496	38.3
Yola	297	22.9
Abakaliki	313	24.2
Location		
Semiurban	502	38.8
Urban	793	61.2
Mother’s age (y)		
<18	106	8.2
19–29	540	41.7
30–39	480	37.1
40–49	113	8.7
>50	22	1.7
Child’s age (mo)		
6–12	541	41.8
13–24	754	58.2
Marital status		
Married	1214	93.7
Cohabiting	5	0.4
Single	21	1.6
Divorced/separated	30	2.3
Widowed	25	1.9
Ethnic group		
Igbo	559	43.2
Yoruba	220	17
Hausa	103	8
Fulani	208	16.1
Kanuri	46	3.6
Others	159	12.3
Highest educational qualification		
None	89	6.9
Primary	63	4.9
Secondary	355	27.4
Tertiary	788	60.8
Occupation		
Civil/public servant	503	38.9
Farmer	88	6.8
Trader	212	16.4
Artisan	123	9.5
Housewife	298	23
Others	71	5.5
The highest educational qualification of the spouse		
None	79	8.1
Primary	21	2.2
Secondary	177	18.2
Tertiary	698	71.6
Occupation of spouse		
Civil/public servant	595	60.6
Farmer	91	9.3
Trader	132	13.4
Artisan	49	5
Others	115	11.7
Type of family		
Monogamy	1071	82.7
Polygamy	220	17
Others	4	0.3
Religion		
Islam	511	52.8
Christianity	450	46.5
Traditional religion	6	0.6
Others	1	0.1
Number of children		
<4	892	68.9
4–6	317	24.5
>7	86	6.6
Number of household members		
<4	248	19.2
4–6	707	54.6
>7	340	26.3
Food insecurity status		
Food secure	437	45.0
Food insecure	535	55.0
Minimum dietary diversity (6–23 mo)		
Low dietary diversity	1073	82.9
Medium dietary diversity	148	11.4
High dietary diversity	74	5.7
Minimum meal frequency (6–23 mo)		
No	599	46.3
Yes	696	53.7

### Dietary intake, food insecurity, and wealth index

**FIGURE 2:**
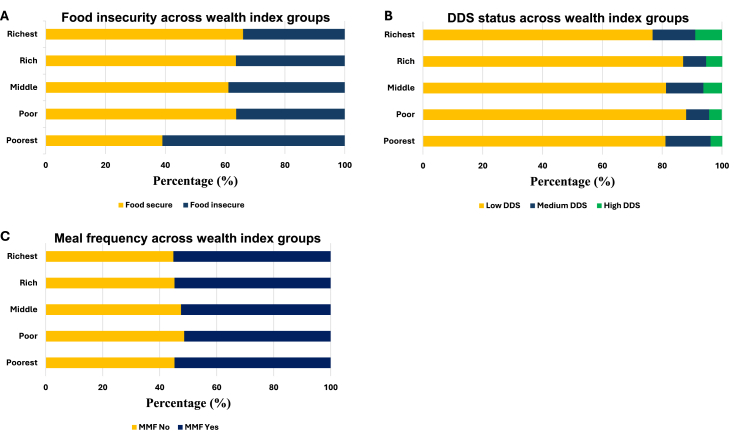
Dietary intake distributions across Household Wealth Index categories. The distribution of (A) food insecurity, (B) minimum dietary diversity, (C) and minimum frequency across household wealth categories. DDS, Dietary Diversity Score; MMF, minimum meal frequency.

Figure 2 shows the distributions across wealth index categories. Food security status across HWI categories. A significant difference (*P* < 0.0001) was observed in Figure 2A, with the poorest households (Bottom 20%) showing the highest proportion of food insecurity (29.5%). In contrast, households in the highest wealth categories had a greater proportion of food-secure participants, suggesting a positive association between wealth and food security status. According to Figure 2B, a significant difference (*P* = 0.006) was observed between the wealth index of households and the dietary diversity of children aged 6–24 mo. The poorest households had the least dietary diversity (13.5%), whereas high dietary diversity was observed in the wealthiest households. In contrast, although not significant (*P* = 0.877), households in the poor (21%) and middle (20.4%) classes had a higher number of individuals who did not meet the food frequency minimum of feeding their 6–23-month-old child/children 4 times per day (Figure 2C). The wealth index was assessed using the HWI indicators presented in Supplementary Table 2.

### Prevalence of DBM

The prevalence of the DBM was presented in Supplementary Figure 1. On the basis of the phenotypes of DBM, the lowest prevalence of DBM was found among mothers who were underweight and had overweight children (1.9%). The DBM phenotype, consisting of overweight mothers having underweight/stunted or wasted children, had a DBM prevalence of 35.5%, whereas overall DBM prevalence was 37.4%.

### Association between dietary intake, socioeconomic characteristics, and DBM

As a preliminary step in isolating the risk factors for DBM, a χ^2^ analysis was done to identify existing associations between food insecurity, socioeconomic characteristics, sociodemographic characteristics, and DBM. Our findings show a significant association (*P* < 0.05) between MDD (*P* < 0.001), MMF (*P* = 0.003), location (*P* < 0.001), ethnic group (*P* < 0.001), religion (*P* = 0.011), study site (*P* < 0.001) (data not shown), and overall DBM (Table 2).

**TABLE 2:** Association between dietary intake, socioeconomic characteristics, and DBM.

Variables	Overall DBM		*P* value	DBM: overweight mother + underweight/stunted/wasted child		*P* value	DBM: underweight mother + overweight child		*P* value
	No	Yes		No	Yes		No	Yes	
Minimum dietary diversity (6–23 mo)			<0.001^1^			<0.001^1^			0.728
Low dietary diversity	503 (81.8)	257 (70)		527 (81.3)	233 (69.8)		736 (77.6)	24 (72.7)	
Medium dietary diversity	72 (11.7)	76 (20.7)		78 (12)	70 (21)		142 (15)	6 (18.2)	
High dietary diversity	40 (6.5)	34 (9.3)		43 (6.6)	31 (9.3)		71 (7.5)	3 (9.1)	
Minimum meal frequency (6–23 mo)			0.003^1^			0.021^1^			0.031^1^
No	263 (42.8)	191 (52)		284 (43.8)	170 (50.9)		433 (45.6)	21 (63.6)	
Yes	352 (57.2)	176 (48)		364 (56.2)	164 (49.1)		516 (54.4)	12 (36.4)	
Food insecurity status			0.524			0.227			0.026^1^
Food secure	280 (45.5)	167 (45.5)		301 (46.5)	146 (43.7)		426 (44.9)	21 (63.6)	
Food insecure	335 (54.5)	200 (54.5)		347 (53.5)	188 (56.3)		311 (46.3)	12 (36.4)	
Wealth index			0.370			0.145			0.365
Poor	244 (39.7)	134 (36.5)		259 (40)	119 (35.6)		363 (38.3)	15 (45.5)	
Middle	123 (20)	68 (18.5)		131 (20.2)	60 (18)		183 (19.3)	8 (24.2)	
Rich	248 (40.3)	165 (45)		258 (39.8)	155 (46.4)		403 (42.5)	10 (30.3)	
Location			<0.001^1^			<0.001^1^			<0.001^1^
Semiurban	84 (13.7)	104 (28.3)		84 (13)	104 (31.1)		188 (19.8)	0 (0)	
Urban	530 (86.3)	263 (71.7)		563 (87)	230 (68.9)		760 (80.2)	33 (100)	
Mother’s age (y)			0.263			0.208			0.293
<18	41 (6.7)	16 (4.4)		44 (6.8)	13 (3.9)		54 (5.7)	3 (9.1)	
19–29	247 (40.2)	166 (45.2)		264 (40.7)	149 (44.6)		396 (41.7)	17 (51.5)	
30–39	256 (41.6)	148 (40.3)		265 (40.9)	139 (41.6)		395 (41.6)	9 (27.3)	
>40	71 (11.5)	37 (10.1)		75 (11.6)	33 (9.9)		104 (11)	4 (12.1)	
Marital status			0.503			0.485			0.558
Married	567 (94.2)	338 (94.4)		597 (94.2)	308 (94.3)		875 (94.3)	30 (93.8)	
Single	35 (5.8)	20 (5.6)		37 (5.8)	18 (5.5)		53 (5.7)	2 (6.3)	
Ethnic group			<0.001^1^			<0.001^1^			0.005^1^
Igbo	143 (23.3)	129 (35.1)		150 (23.1)	122 (36.5)		265 (27.9)	7 (21.2)	
Yoruba	125 (20.3)	74 (20.2)		138 (21.3)	61 (18.3)		186 (19.6)	13 (39.4)	
Hausa	202 (32.8)	105 (28.6)		138 (33)	93 (27.8)		295 (31.1)	12 (36.4)	
Others	145 (23.6)	59 (16.1)		146 (22.5)	58 (17.4)		203 (21.4)	1 (3)	
Mother’s education			0.383			0.809			0.163
None	48 (7.8)	31 (8.4)		51 (7.9)	28 (8.4)		76 (8)	3 (9.1)	
Primary	37 (6)	22 (6)		39 (6)	20 (6)		57 (6)	2 (6)	
Secondary	172 (28)	120 (32.7)		187 (28.9)	105 (31.4)		277 (29.2)	15 (45.5)	
Tertiary	358 (58.2)	194 (52.9)		371 (57.3)	181 (54.2)		539 (56.8)	13 (39.4)	
Mother’s occupation			0.053			0.607			0.392
Civil/public servant	251 (40.8)	125 (34.1)		264 (40.7)	112 (33.5)		363 (38.3)	13 (39.4)	
Farmer	37 (6)	36 (9.8)		38 (5.9)	35 (10.5)		72 (7.6)	1 (3)	
Trader	68 (11.1)	48 (13.1)		71 (11)	45 (13.5)		113 (11.9)	3 (9.1)	
Artisan	58 (9.4)	46 (12.5)		59 (9.1)	45 (13.5)		103 (10.9)	1 (3)	
Housewife	158 (25.7)	84 (22.9)		171 (26.4)	71 (21.3)		229 (24.1)	13 (39.4)	
Others	43 (7)	28 (7.6)		45 (6.9)	26 (7.8)		69 (7.3)	2 (6.1)	
Spouse’s education			0.438			0.423			0.288
None	47 (7.7)	32 (8.8)		50 (7.8)	29 (8.8)		76 (8.1)	3 (9.1)	
Primary	14 (2.3)	7 (1.9)		16 (2.5)	5 (1.5)		19 (2)	2 (6.1)	
Secondary	103 (16.8)	74 (20.4)		110 (17.1)	67 (20.3)		170 (18)	7 (21.2)	
Tertiary	448 (73.2)	250 (68.9)		469 (72.7)	229 (69.4)		677 (71.9)	21 (63.6)	
Spouse’s occupation			0.258			0.133			0.694
Civil/public servant	382 (62.1)	213 (58)		402 (62)	193 (57.8)		575 (60.6)	20 (60.6)	
Farmer	58 (9.4)	33 (9)		60 (9.3)	31 (9.3)		89 (9.4)	2 (6.1)	
Trader	72 (11.7)	60 (16.3)		75 (11.6)	57 (17.1)		129 (13.6)	3 (9.1)	
Artisan	28 (4.6)	21 (5.7)		30 (4.6)	19 (5.7)		47 (5)	2 (6.1)	
Others	75 (12.2)	40 (10.9)		81 (12.5)	34 (10.2)		109 (11.5)	6 (18.2)	
Type of family			0.457			0.402			0.460
Monogamy	489 (79.5)	290 (79)		516 (79.6)	263 (78.7)		752 (79.2)	27 (81.8)	
Polygamy	126 (20.5)	77 (21)		132 (20.4)	71 (21.3)		197 (20.8)	6 (18.8)	
Religion			0.011^1^			<0.001^1^			0.005^1^
Islam	316 (51.4)	154 (42)		341 (52.6)	129 (38.6)		445 (46.9)	25 (75.8)	
Christianity	295 (48)	210 (57.2)		303 (46.8)	202 (60.5)		497 (52.4)	8 (24.2)	
Traditional religion	4 (0.7)	3 (0.8)		4 (0.6)	3 (0.9)		7 (0.7)	0 (0)	
Number of children			0.181			0.071			0.464
<4	398 (64.7)	227 (61.9)		422 (65.1)	203 (60.8)		601 (63.3)	24 (72.7)	
4–6	159 (25.9)	113 (30.8)		165 (25.5)	107 (32)		266 (28)	6 (18.2)	
>7	58 (9.4)	27 (7.4)		61 (9.4)	24 (7.2)		82 (8.6)	3 (9.1)	
Number of household members			0.312			0.414			0.868
<4	119 (19.3)	65 (17.7)		125 (19.3)	59 (17.7)		178 (18.8)	6 (18.2)	
4–6	294 (47.8)	194 (52.9)		312 (48.1)	176 (52.7)		470 (49.5)	18 (54.5)	
>7	202 (32.8)	108 (29.4)		211 (32.6)	99 (29.6)		301 (31.7)	9 (27.3)	

Abbreviation: DBM, double burden of malnutrition. The terms “overweight mother” and “overweight child” encompass both overweight and obese categories.

1 Statistically significant associations *P* < 0.05. Fisher’s exact test was used to assess significant associations.

### Interaction effects of wealth index, location, and dietary intake on DBM

**FIGURE 3:**
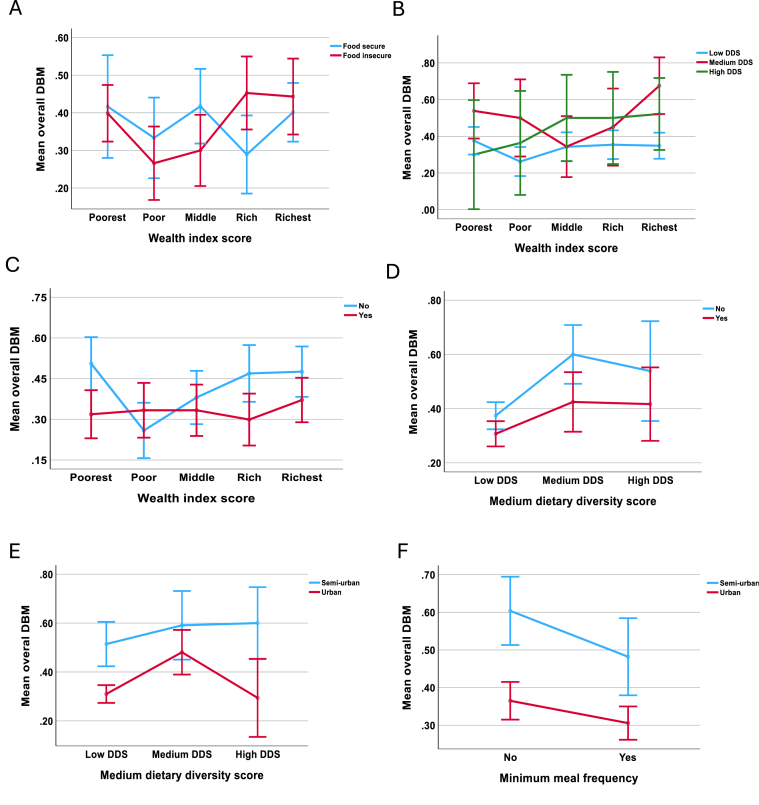
DBM trends across socioeconomic characteristics by dietary intake parameters. (A) Interaction between food insecurity and wealth index in relation to DBM, (B) interaction between MDD and wealth index in relation to DBM, (C) interaction between MDD and maternal education in relation to DBM, (D) interaction between MMF and wealth index in relation to DBM, (E) interaction between MMF and maternal education in relation to DBM, and (F) interaction between MMF and MDD in relation to DBM. DBM, double burden of malnutrition; DDS, Dietary Diversity Score; MDD, minimum dietary diversity; MMF, minimum meal frequency.

Emerging literature has reported that socioeconomic characteristics and dietary intake are strong risk factors for DBM [30]. According to Figure 3A, a higher DBM prevalence was observed among food-secure households in higher wealth categories, although this trend was not significant (*F* value = 1.84, *P* = 0.058). The interaction between wealth index and MDD (wealth × MDD) revealed that the richest households with the highest MDD had a higher tendency to DBM (*F* value = 2.49, *P* = 0.002) (Figure 3B). The poorest families, who did not meet the MMF, showed a higher tendency to DBM than those from the other wealth categories (MMF × wealth) (*F* value = 2.77, *P* = 0.003) (Figure 3C). In addition, households that did not meet the MMF requirement (*F* value = 5.80, *P* < 0.001) but had a medium DDS showed a higher pattern of DBM than those who met the requirement (Figure 3D). Households in semiurban areas with high MDD (*F* value = 9.13, *P* < 0.001) and those not meeting the MMF (*F* value = 12.78, *P* < 0.001) had a higher risk of DBM (Figure 3E, F).

### Risk of DBM

Table 3 presents the sociodemographic risk factors of DBM assessed using GEE analysis. The main findings from the adjusted model suggest that not meeting the MMF (*P* = 0.029), even when food secure (*P* < 0.001), is associated with a higher risk of having DBM. Participants residing in the semiurban areas had a 2-fold risk of having DBM. Similarly, in the unadjusted model, not meeting the MMF and residing in the semiurban areas were associated with a higher risk of DBM.

**TABLE 3:** Dietary and sociodemographic risk factors of DBM.

	OR (95% CI)	*P* value	AOR (95% CI)	*P* value
Minimum dietary diversity (6–23 mo)				
Low dietary diversity	0.60 (0.30, 1.22)	0.161	0.76 (0.59, 0.97)	0.026^1^
Medium dietary diversity	1.24 (0.69, 2.25)	0.474	1.46 (0.83, 2.56)	0.185
High dietary diversity	—		—	
Minimum meal frequency (6–23 mo)				
No	1.45 (1.03, 2.03)	0.032^1^	1.41 (1.04, 1.92)	0.029^1^
Yes	—		—	
Food insecurity status				
Food secure	1.00 (0.86, 1.15)	0.958	1.22 (1.09, 1.37)	<0.001^1^
Food insecure	—		—	
Location				
Semiurban	2.50 (2.05, 3.04)	<0.001^1^	2.11 (1.93, 2.30)	<0.001^1^
Urban	—		—	
Ethnic group				
Igbo	2.23 (1.19, 4.20)	0.013^1^	1.19 (0.90, 1.56)	0.218
Yoruba	1.46 (0.94, 2.26)	0.096	1.46 (0.90, 2.37)	0.126
Hausa	1.28 (0.85, 1.91)	0.234	1.43 (0.81, 2.51)	0.218
Others	—		—	
Religion				
Islam	0.65 (0.10, 4.22)	0.652	1.12 (0.30, 4.21)	0.871
Christianity	0.95 (0.22, 4.07)	0.952	1.44 (0.39, 5.32)	0.586
Traditional religion	–		–	

Abbreviations: AOR, adjusted odds ratio; CI, confidence interval; DBM, double burden of malnutrition; OR, odds ratio.

1 Statistically significant associations *P* < 0.05.

## Discussion

This study examined the factors influencing the DBM among mother–child pairs across 4 states in Nigeria. The prevalence of DBM and food insecurity-related factors associated with it was also examined. Findings revealed a DBM prevalence of 37.4%, with the most common pattern being overweight mothers with underweight, stunted, or wasted children (34%). After accounting for cluster-level correlations, we found that DBM remains a significant nutritional problem in food-secure suburban communities, and several dietary factors were strongly linked to its occurrence.

Compared with results from studies conducted in Malawi (5.5%) [31], Nepal (6.6%) [32], Bangladesh (6.3%) [33], India (6%) [1], Ethiopia (1.65%) [34], and Malaysia [35], the prevalence of DBM observed in our study was significantly higher. This elevated burden may reflect the unique nutrition transition occurring in many Nigerian settings, signaling a rapid shift in dietary patterns, inconsistent feeding practices, and food quality.

A strong association between MMF and DBM was observed in our study. Children who did not meet the recommended MMF had a higher risk of experiencing the DBM. This is in agreement with evidence suggesting that inadequate feeding frequency may contribute to poor growth while simultaneously increasing the risk of excessive weight gain in later life [36,37]. In many low-income households, children who miss structured meals often rely on cheap, energy-dense snacks, which may explain this pattern [38]. Our findings highlight that ensuring frequent and appropriately timed meals remains central to preventing DBM.

Appropriate dietary diversity is crucial for DBM prevention [39]. The adjusted model showed that lower dietary diversity was associated with a lower risk of DBM. Although our findings are in contrast to several studies [[40], [41], [42]] similar results have been reported in African settings, which may be a result of increased processed foods found in most food groups in recent years [28]. Dietary diversity may encompass ultraprocessed, energy-dense foods, especially within urbanizing contexts [[43], [44], [45]]. Studies suggest DBM is impacted by transitions toward calorie-dense but low-nutrient diets, urbanization, and socioeconomic disparity [46]. In Nigeria, traditional dietary patterns increased both underweight and overweight/obesity, whereas diversified dietary patterns decreased [47]. This reflects our finding that diversity alone does not guarantee nutritional adequacy if it includes unhealthy foods. This paradox highlights the changing nutrition landscape where diverse diets may not always equal nutritious diets, especially in environments undergoing rapid nutrition transition. We also found that food-secure households had higher odds of DBM compared with moderately or severely food-insecure households. Although surprising, this pattern has been documented in settings where wealthier households have shown a 44% higher likelihood of experiencing DBM [30].

Households living in semiurban areas had significantly higher DBM risk compared with those in urban areas. Semiurban settings are often characterized by rapid dietary shifts, increased availability of ultraprocessed foods, and limited nutrition education [48]. These environments are prone to greater availability and marketing of ultraprocessed products, which may lead to excessive caloric intake, but may still struggle with deprivation that sustains undernutrition. The strong effect of location on our clustered model emphasizes the importance of designing context-specific interventions rather than relying solely on national-level strategies.

The interaction model findings in our study revealed how dietary and socioeconomic factors jointly shape DBM. The interaction between dietary diversity and wealth suggests that the impact of MDD varies across economic groups. Wealthier households may achieve dietary diversity through more processed foods, whereas poorer households may do so through traditional staples. The interactions involving MMF further highlight that feeding frequency does not operate in isolation but is influenced by both the household's economic capacity and the child’s overall feeding pattern. These complexities emphasize that DBM is not driven by single behaviors but by the broader food environment in which families make daily feeding decisions.

Overall, our findings show that addressing DBM in Nigeria requires a dual strategy of promoting consistent meal frequency and emphasizing the quality of dietary diversity rather than diversity alone. Public health messaging should highlight the risks of energy-dense processed foods, even in households that appear food-secure. Interventions must also be sensitive to the rapid nutrition transition occurring in semiurban areas. Our findings also challenge the long-held belief that poverty and food insecurity are the dominant drivers of DBM and instead highlight the importance of diet quality, especially in semiurban households. These findings call for interventions targeted at nutrition education in rapidly urbanizing communities where DBM patterns are shifting. This research was not without limitations. Although missing data were handled using pairwise deletion, we acknowledge that pairwise deletion assumes data are missing completely at random, and residual bias cannot be entirely ruled out. In addition, the study’s cross-sectional design limits the ability to establish causal relationships between the identified factors and DBM outcomes. Furthermore, our study did not include participants from diverse community settings and truly rural areas, which may limit the generalizability of findings to less urbanized populations. Therefore, future research should involve larger, more representative samples and be conducted across all 6 geopolitical zones of Nigeria to enable deeper exploration of sociocultural influences on DBM.

In conclusion, DBM among mother–child pairs in Nigeria is shaped by a complex interplay of urban-semiurban disparities, socioeconomic determinants, and evolving dietary patterns. The high prevalence of overweight mothers with undernourished children reflects an ongoing nutrition transition, in which limited access to diverse, nutrient-rich foods coexists with increased consumption of energy-dense, nutrient-poor options. Addressing this dual challenge requires context-specific strategies that account for the distinct drivers of DBM across settings. Interventions should prioritize the design and implementation of culturally relevant, location-specific nutrition programs that not only improve meal frequency and dietary diversity but also enhance overall diet quality in semiurban areas.

## Author contributions

The authors’ responsibilities were as follows – GOI: conceptualized, acquired the fund, and supervised the research; BFO, GOI: contributed to data curation and methodology; HI, AAF, LOE, IME: contributed to data collection; BFO: contributed to formal analysis and project administration; BFO, FAA, CJE, ON: contributed to original draft writing; BFO, FAA: contributed to data visualization; BFO, FAA, CJE, ON, HI, AAF, LOE, GOI: reviewed and edited the final draft; and all authors: read and approved the final manuscript.

## Data availability

The datasets used and/or analyzed during the current study are available from the corresponding author on reasonable request.

## Funding

All authors are members of the Pediatric Nutrition Working Group, supported by Nutrition Drive for Healthy Diet Initiative under the cooperative agreement (OB/PN-2022-0103). The funders supported the conceptualization and validation of the research design.

## Declaration of Generative AI and AI-assisted technologies in the writing process

The author(s) declare that no generative AI or AI-assisted technologies were used in the writing of this manuscript.

## Conflict of interest

The authors report no conflicts of interest.
