# Supplementary material for: Location-Specific Predictors of Double Burden of Malnutrition among Nigerian Mother–Child Pairs: Re-evaluating Dietary Quality and Socioeconomic Factors
Source: J Nutr. 2026 Mar 12;156(5):101478. doi: 10.1016/j.tjnut.2026.101478 (PMC13197940; doi:10.1016/j.tjnut.2026.101478)
Supplement: multimedia component 1 [file mmc1.pdf]

Location-specific predictors of double burden of malnutrition among Nigerian Mother-Child pairs: Re-evaluating dietary quality and socioeconomic factors.

First author: Beulah F. Ortutu

Supplementary data

Supplementary Table 1. Household wealth index responses of participants

More than half of the mother-child pairs lived as tenants (51%) in 1–3-bedroom apartments (52.5%). The majority did not have a car (53.1%) or motorcycle (56.9%), cable (50.5%), computer set (65.5%), or a washing machine (74.1%).

Supplementary Table 1. Household wealth index

Variables	Frequency	Percentage (%)	Component weight (PC1)
**Form of house ownership**			0.002
Tenant	660	51	
Family house	290	22.4	
House owner/landlord	345	26.6	
**Type of apartment**			0.488
Single room, single room, and living room, self-contained room	161	12.5	
1–2-bedroom flats	680	52.5	
3-bedroom flats, duplex, mansion, and above	454	35.1	
**Material (wall) of the house**			0.631
Cane/palm trunks, no wall, bamboo with mud	32	2.5	
Stone with mud, wood/planks	85	6.6	
Blocks, cement, bricks	1178	91	
**Type of lighting**			0.682
No electricity (candle, lantern, torchlight)	72	5.5	
Electricity (PHCN/NEPA)	690	53.3	
PHCN + alternate lighting source	533	41.2	
**Type of cooking fuel**			0.344
Firewood, shrubs/grass crops residue	102	10.4	
Coal, Lignite, kerosene	211	21.5	
Gas, electricity	668	68.1	
**Number of people who sleep in a room**			0.463
>5 persons	105	8.1	

3-5 persons	408	31.5	
<3 persons	782	60.4	
**Sources of drinking water**			0.731
Unprotected well or pond	58	4.4	
Protected well, tap water, borehole	461	35.6	
Regularly treated borehole, sachet/bottled water	776	59.9	
**Toilet facility**			0.721
Open pit latrine	96	7.4	
Closed pit latrine of flush/ water system toilet shared with another household	228	17.6	
Water system/flush toilet	971	75	
**Mobile phone**			0.343
No	31	2.4	
Yes	1264	97.6	
**Refrigerator**			0.588
No	285	22	
Yes	1010	78	
**Motorcycle**			-0.101
No	996	76.9	
Yes	299	23.1	
**Car**			0.535
No	687	53.1	
Yes	608	46.9	
**Television**			0.564
No	171	13.2	
Yes	1124	86.8	
**Bank account**			0.484
No	183	14.1	
Yes	1112	85.9	
**Cable**			0.503
No	654	50.5	
Yes	641	49.5	
**Computer/laptop**			0.501
No	848	65.5	

Yes	447	34.5	
**Washing machine**			0.410
No	960	74.1	
Yes	335	25.9	
**Shop/farm**			-0.008
No	891	68.8	
Yes	404	31.2	
**Domestic servant**			0.200
No	799	61.7	
Yes	167	12.9	
**Extra landed property/building**			0.340
No	634	65.8	
Yes	330	34.2	

PHCN: Power Holding Company of Nigeria; NEPA: National Electric Power Authority

Supplementary Table 2. Household Food Insecurity Access Scale (HFIAS) responses of participants

About 1.2% of the participant households often worried that the household would not have enough food, while 1.8% consumed less preferred food, and 2.4% consumed a limited variety of food.

Supplementary Table 1. Household Food Insecurity Access Scale

Variables	Frequency	Percentage (%)
**Worried that the household would not have enough food**		
None	629	65.6
Rarely (1-2 times last month)	242	24.8
Sometimes (3-10 times last month)	90	9.2
Often (>10 times last month)	16	1.4
**If you or household members were not able to eat the kinds of foods preferred because of a lack of resources**		
None	622	63.6
Rarely (1-2 times last month)	254	26
Sometimes (3-10 times last month)	84	8.6
Often (>10 times last month)	18	1.8
**If you or household members eat a limited variety of food due to a lack of resources**		
None	595	60.9
Rarely (1-2 times last month)	260	26.6
Sometimes (3-10 times last month)	99	10.1

Often (>10 times last month)	23	2.4
**If you or any household member had to eat some foods that you preferred not to eat because of a lack of resources to obtain other types of food**		
None	635	65
Rarely (1-2 times last month)	226	23.2
Sometimes (3-10 times last month)	95	9.7
Often (>10 times last month)	20	2
**If you or any household member had to eat smaller meals than you felt you needed, because there was not enough food**		
None	718	73.7
Rarely (1-2 times last month)	163	16.7
Sometimes (3-10 times last month)	80	8.2
Often (>10 times last month)	13	1.3
**If you or any household member had to eat fewer meals in a day because there was not enough food**		
None	727	74.6
Rarely (1-2 times last month)	170	17.5
Sometimes (3-10 times last month)	64	6.6
Often (>10 times last month)	13	1.3
**If there was no food of any kind to eat in your household because of resources to get food**		
None	811	83.2
Rarely (1-2 times last month)	105	10.8
Sometimes (3-10 times last month)	49	5
Often (>10 times last month)	9	0.9
**If you or any household member goes to sleep at night hungry because there was not enough food**		
None	857	87.8
Rarely (1-2 times last month)	74	7.6
Sometimes (3-10 times last month)	37	3.8
Often (>10 times last month)	8	0.8
**If you or any household member goes a whole day and night without eating anything because there was not enough food**		
None	863	88.4
Rarely (1-2 times last month)	72	7.4
Sometimes (3-10 times last month)	34	3.5
Often (>10 times last month)	7	0.7

Supplementary Table 3. Dietary diversity food group classification

Food group	Frequency	Percentage (%)
**Grains, roots, and tubers (Yam, potatoes, garri/fufu, rice, maize, pasta)**		
No	948	73.2
Yes	347	26.8
**Legumes and nuts (Beans, groundnut, soyabeans, fiofio, okpa, odudu)**		
No	1059	81.8
Yes	236	18.2
**Dairy products (Milk, cheese, yoghurt, infant formula, breastmilk)**		
No	999	77.1
Yes	296	22.9
**Fleshy food (Meat, fish, poultry/chicken, liver/organ meat etc)**		
No	1152	89.0
Yes	143	11.0
**Eggs**		
No	1171	90.4
Yes	124	9.6
**Vitamin A-rich fruit and vegetables (carrot, ugu, tomatoes, onion, green pepper, paw-paw)**		
No	1116	86.2
Yes	179	13.8
**Other fruits and vegetables (banana, oranges, pineapple, watermelon etc)**		
No	1110	85.7
Yes	185	14.3
**Sweet beverage (Softdrinks, fruit drinks-bobo, fanta, chivita, caprisonne etc)**		
No	1192	92.0
Yes	103	8.0

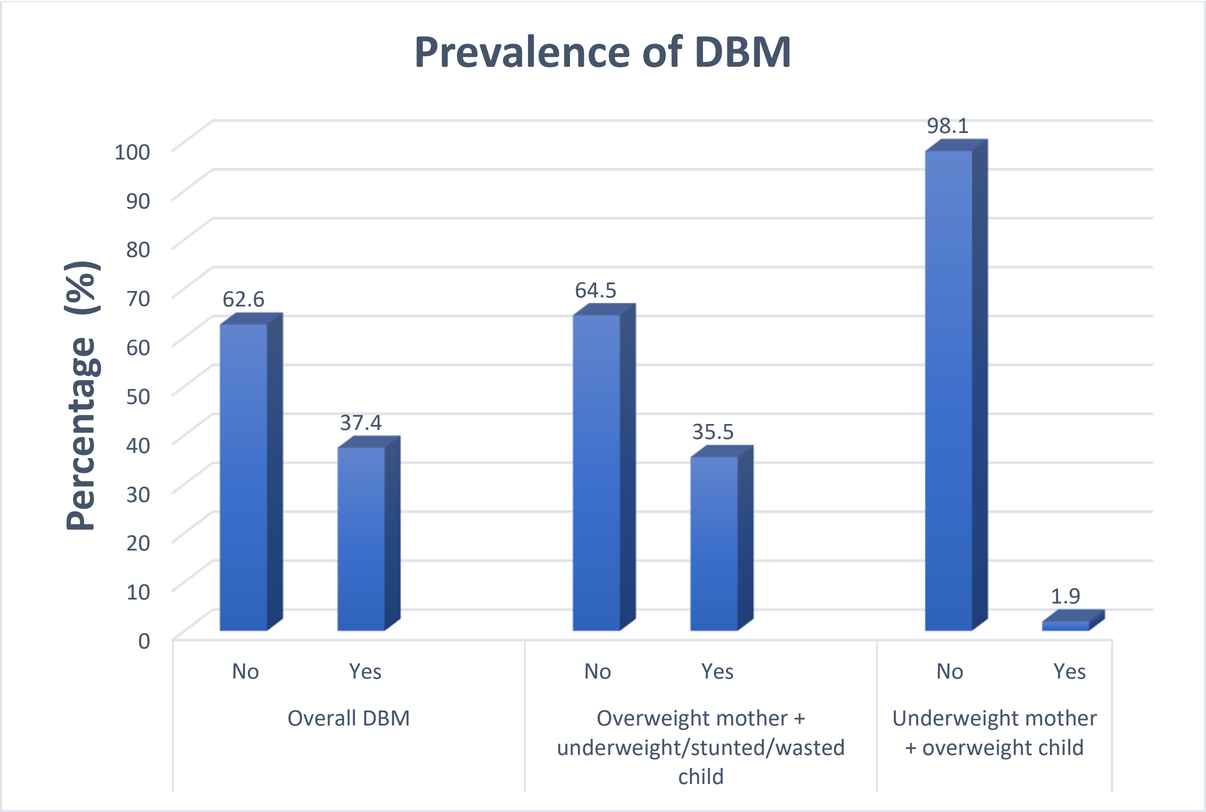

Supplementary Figure 1. Prevalence of double burden of malnutrition.

DBM: double burden of malnutrition
